# Impact of Climate and Hydrological Variability on Drinking Water Production and Trihalomethane Levels: A Case Study in Barcelona, Spain (2010–2024)

**DOI:** 10.1021/acsestwater.5c01024

**Published:** 2025-11-25

**Authors:** Fang Fang Chen Chen, Pere Emiliano, Fernando Valero, Xavier Basagaña, Cristina M. Villanueva

**Affiliations:** † 310844 ISGlobal, Doctor Aiguader 88, Barcelona 08003, Spain; ‡ Universitat Pompeu Fabra (UPF), Doctor Aiguader 88, Barcelona 08003, Spain; § Ens d’Abastament d’Aigua Ter Llobregat (ATL), Sant Martí de l′Erm, 2., 08970 Sant Joan Despí, Barcelona, Spain; ∥ CIBER Epidemiologia y Salud Pública (CIBERESP), Instituto de Salud Carlos III, Madrid 28029, Spain; ⊥ Hospital del Mar Medical Research Institute, Doctor Aiguader 88, Barcelona 08003, Spain

**Keywords:** THM, water quality, extreme weather events

## Abstract

Surface water-based utilities increasingly face challenges in drinking water production during prolonged droughts and heavy rainfall events. We assessed the impact of climate and hydrological variability on trihalomethane (THM) levels in two drinking water treatment plants in Barcelona: one river-based (Llobregat plant) and one reservoir-based (Ter plant). We examined data from 15 years (2010–2024) using generalized additive models (GAMs) to evaluate the change (β) in chloroform, bromodichloromethane, dibromochloromethane, bromoform, and total THMs (THM4), by extreme (≤percentile 10, ≥percentile 90) hydrometeorological predictors, including temperature, river flow, or reservoir level relative to normal conditions (P10–P90), and the Standardized Precipitation Evapotranspiration Index (SPEI 1). In the Llobregat plant, THMs were unaffected under low river flow events (≤P10), while THM4 decreased by −1.41 (confidence interval (CI) 95%: −2.77, −0.05) during high river flow events (≥P90), mainly driven by bromoform (β: −2.64, CI 95%: −3.61, −1.67). In the Ter plant, THM4 increased by 1.64 (CI 95%:0.09, 3.19) and 4.08 (CI 95%:0.83, 7.33), respectively, under high (≥P90) and low (≤P10) reservoir levels. Overall, moderate effects of extreme weather events on THM levels were observed, attributed to climate-resilient water management strategies. Further research is needed in other settings with diverse water sources and management.

## Graphical abstract

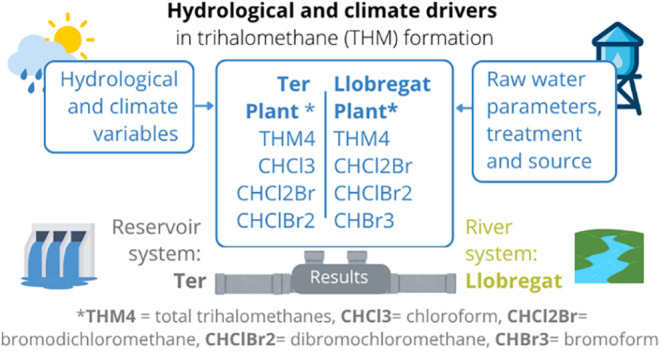

## Introduction

Access to safe drinking water is a fundamental human right and an essential need^1^ under pressure by global change. There is growing evidence showing the impacts of climate change on the water cycle^2,3^ and water quality at the source.^4–6^ However, the effects on finished drinking water quality remain poorly understood. Climate-related degradation of drinking water quality has been suggested,^7^ leading to potential health risks. Nevertheless, evidence of how extreme weather events affect chemical quality in treated drinking water is limited.

The Mediterranean region is a climate change hot-spot, faces rising population density, decreasing precipitation, and a growing risk of aridification.^8,9^ The 2021–2023 period has been reported as the driest since 1835.^10,11^ Extreme weather events are expected to become more frequent according to future climate projections.^11^ In Barcelona, severe droughts in 2008 and from 2022 to 2024,^12,13^ as well as major floods in 2005 and 2020,^14,15^ have tested the resilience of drinking water treatment plants. These events impact watershed sustainability and source water quality, placing additional pressure on limited water.^11,16,17^

Disinfection is crucial in controlling microbiological contamination. It has been a central public health intervention since the early 20th century.^18,19^ However, disinfection leads to the formation of disinfection byproducts (DBPs) when disinfectants react with natural organic matter or inorganic ions like bromide in raw water.^20–22^ Among more than 800 identified DBPs, trihalomethanes (THM) are the most prevalent chlorination DBPs.^20,23^ THM are regulated due to their association with health risks, such as bladder cancer.^24,25^ In particular, brominated THMs (bromodichloromethane, CHCl2Br; dibromochloromethane, CHClBr2; bromoform, CHBr3) are more genotoxic compared to chlorinated analogues (chloroform, CHCl3).^26^ The regulatory limit for THM is 80 μg/L in the USA, 100 μg/L in Europe,^27,28^ and varies worldwide.^28–30^

THM formation is influenced by climate-sensitive factors and seasonal fluctuations such as temperature and total organic carbon (TOC), along with source water characteristics.^31–33^ Surface water typically contains a higher TOC than groundwater, leading to higher THM formation. Seawater minimally forms THMs because of low TOC. However, when desalinated seawater (containing bromide) is mixed with other water sources, bromide speciation may change, leading to the formation of brominated THMs.^23^ Other factors, such as pH and temperature, also play a significant role in THM formation.^29^

This study investigates the impact of extreme weather and hydrological events on THM levels in finished drinking water in Barcelona, Spain, where advance treatment technologies have been implemented to manage THM formation.^34^ Specifically, we explored the relationship between source water availability, temperature, and drought conditions on THM concentrations at two plants using different sources and treatment processes from 2010 to 2024.

## Materials and Methods

### Study Area

The metropolitan area of Barcelona has over 5.5 M inhabitants located on the Northeast Spanish Mediterranean coast.^35^ Mediterranean climate is characterized by mild, humid winters and dry, hot summers,^16^ experiencing two rainy seasons (spring, autumn) that can cause flash floods. The main drinking water sources are the Llobregat and Ter Rivers,^16^ treated at three plants: Ter (Cardedeu), Llobregat (Abrera), and Sant Joan Despí. This study focuses on the two plants managed by the public company Ens d’abastament d’Aigua Ter Llobregat (ATL): Llobregat and Ter plants (Figure 1). Since 2010, the two rivers have been interconnected to optimize the water supply. Additionally, desalinated seawater, coming from the El Prat plant since 2009 and from the Tordera plant since 2002, supplements, respectively, the Llobregat and Ter plants.

**Figure 1:**
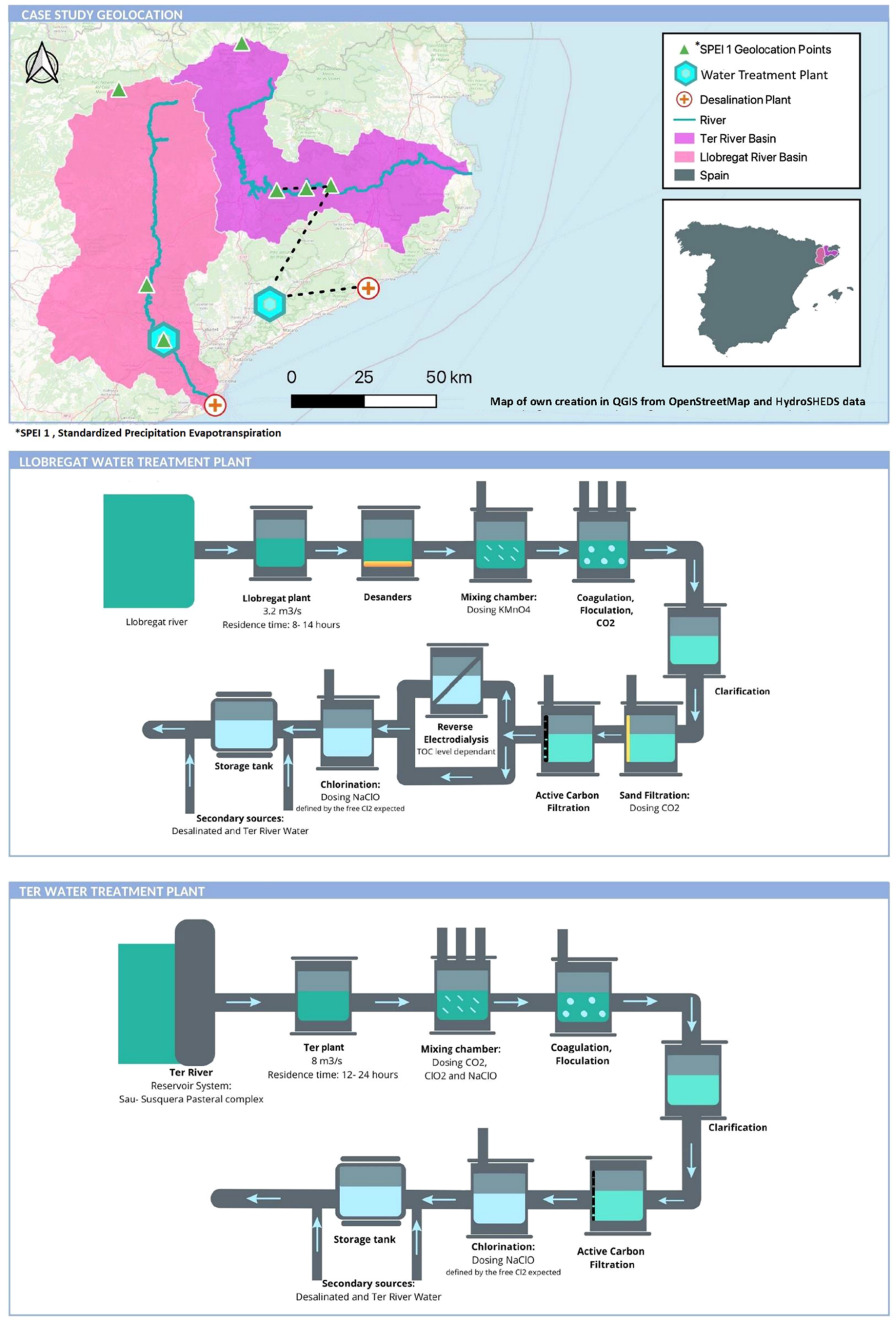
Study area and drinking water treatment plants operational system.

The Llobregat basin′s north and central areas include pine forests, agriculture, industry, and potash mines,^12^ which increase river salinity and bromide levels.^16,36^ To reduce brominated THMs, an electrodialysis reversal (EDR) system was installed at the Llobregat plant in 2009.^23,37^ In contrast, the Ter basin is dominated by riparian forests.^38^ Ter River′s flow is regulated by a system of three reservoirs (Sau, Susqueda, and Pasteral), which help reduce treatment needs by acting as artificial sediment cells, reducing the need for extensive treatment.^39^ Sau and Susqueda deliver water after selecting the best layer into the Pasteral reservoir. Ter plant is directly supplied by the Pasteral reservoir.

### Water and Climate Data

ATL provided data from 2010 to 2024 for Llobregat and Ter plants, including concentrations of four trihalomethane species and total trihalomethanes (THM4, μg/L) measured at the plant outlets. Raw water parameters measured at the inlet included river flow (m^3^/s) or reservoir volume (hm^3^), temperature (°C), conductivity (μS/cm), ammonia (mg/L), nitrite (mg/L), nitrate (mg/L), pH, TOC (mg/L), ultraviolet absorbance (UV Abs, abs/m), and bromide (mg/L). Monitoring was conducted every 4–5 days for Llobregat and weekly for Ter. Operational data included the percentage of water treated by EDR at Llobregat and the proportion of water from secondary sources, such as desalinated water, for both plants.

We also analyzed the Standardized Precipitation Evapotranspiration Index (SPEI), a validated drought and wetness indicator from the Spanish National Research Council.^40^ SPEI 1 values were weekly medians from key locations of each basin (Figure 1). Then, we merged it with water quality data using a 1-week lag to account for delayed THM responses. The SPEI ranges from severely wet, values ≥1.5, while ≤−1.5 indicates severe drought.

### Statistical Analyses

We explored how extreme weather events influence THM formation using two approaches (Figure 2): one based on temperature and hydrological indicators (river flow (m^3^/s) for Llobregat plant, and reservoir volume (hm^3^) for Ter plant), and another using the SPEI 1 as a marker for drought and wetness. Analyses were done separately for each plant due to differences in catchments and treatment.^23^ Data below detection limits were imputed as half the detection limit, and duplicates were removed. To avoid multicollinearity, we selected representative water quality variables consistent across plants based on the Spearman correlations plot (Supporting Figure 1). Final models were developed according to Figure 2 and the following regression models.

$$\mathrm{THM}={\beta_{a}}^{*}s\left(\mathrm{time}\right)+{\beta_{b}}^{*}p\mathrm{redictors}+{\beta_{c}}^{*}c\mathrm{ovariates}+{\beta_{d}}^{*}s\mathrm{ource}$$

where

**Figure 2:**
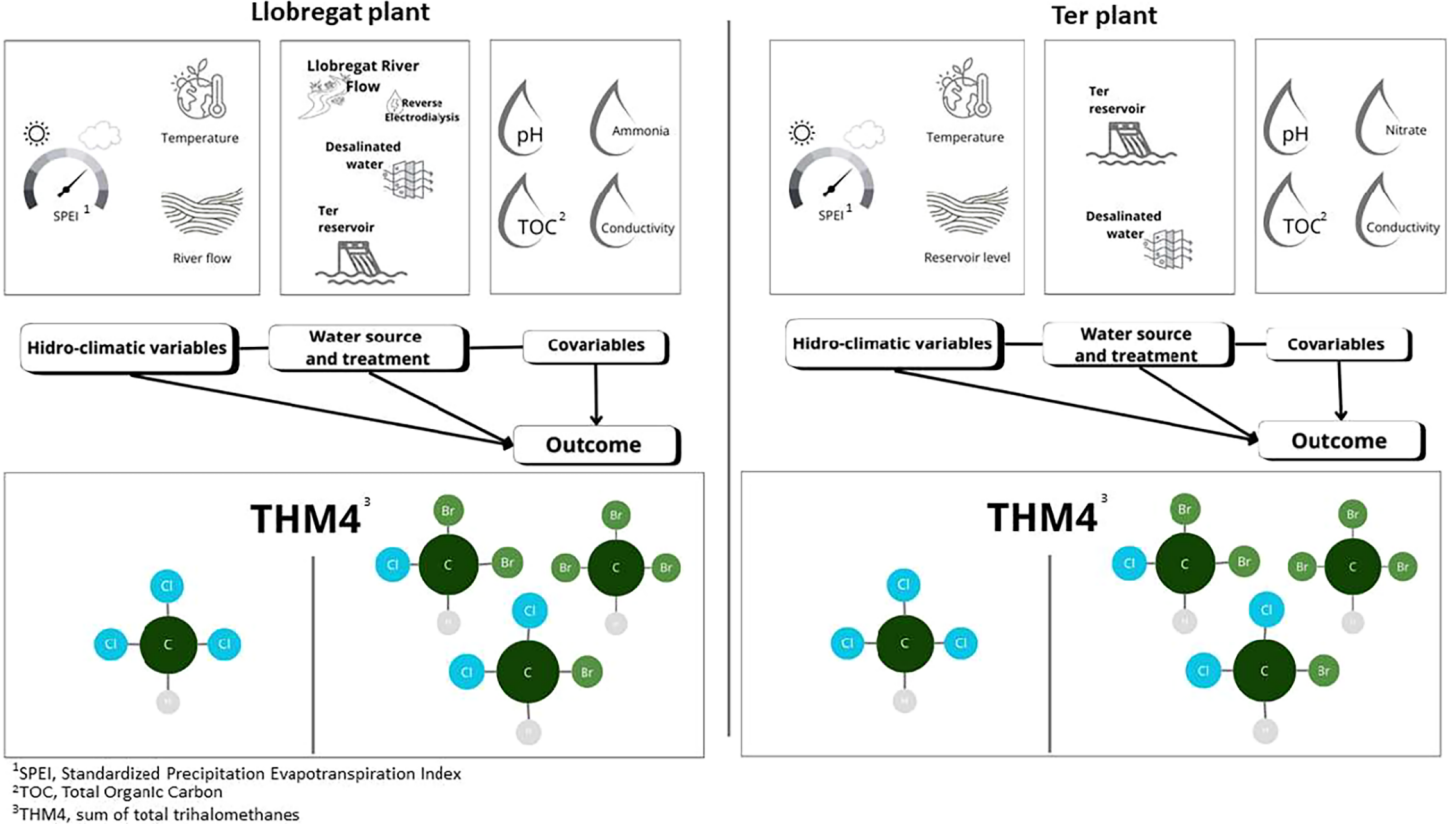
Conceptual model for both drinking water treatment plants.

**β**_**a**_, **β**_**b**_, **β**_**c**_, **and β**_**d**_: estimated regression coefficients corresponding to each variable group.

**THM:** represents THM4, CHCl3, CHCl2Br, CHClBr2, or CHBr3.

**s­(time):** a 60-basis spline function of time, in a generalized additive models (GAMs) model was used to capture the nonlinear temporal pattern of seasonality and trend.

**Predictors:** temperature and Llobregat River flow (for Llobregat plant) or Sau–Susqueda–Pasteral reservoir volume (for Ter plant). The SPEI 1 index is used in separate models.

**Covariates:** conductivity, ammonia or nitrate, pH, and TOC. For the Llobregat plant, the percentage of water treated by EDR is also included.

**Source:** % Llobregat water (in Llobregat plant); % Ter water (in Ter plant).

Separate models were built for each THM species and the total THM using generalized additive models (GAMs) to explore nonlinear associations. Chloroform was excluded from the Llobregat model and bromoform from the Ter model due to consistently negligible levels. Models using continuous predictors incorporated cubic regression splines on river flow or reservoir volume (*k* = 2) and time (*k* = 60) to account for seasonal and long-term trends. Models were also run using predictors (temperature and water flow or volume) in categories based on the 10th and 90th percentiles (P10, P90), included as dummy variables. To control for potential confounding, we adjusted all models for a consistent set of water quality covariates across both plants (Figure 2). To explore potential effect modification, we stratified models by water source and, in the case of the Llobregat plant, also by EDR-treated water.

Finally, SPEI 1 was analyzed as a categorical variable using a ≥ |1.5| threshold to identify extreme drought or wetness. These models included the same covariates, a time spline, and stratification by water source. Lagged effects were explored but showed no improvement in the model fit. Nevertheless, a 1-week lag was applied when merging SPEI 1 data to account for temporal delays in THM responses. All analyses were conducted using statistical software R Studio version 4.4.1, and the spatial data processing was conducted in QGIS 3.22.

## Results

### Hydrological Trends in Ter and Llobregat Plants

Table 1 summarizes the main variables under study, and Figure 3 depicts the temporal variation. The Llobregat River flow had a median of 8.3 m^3^/s with notable variability (interquartile range (IQR): 6.5–11.8 m^3^/s). In the Ter basin, the reservoir volume showed moderate fluctuation (IQR: 217–348 hm^3^), with a median of 305 hm^3^. Over the 15-year period, THM level declined by approximately 20 μg/L (Figure 3 (1,2)), while raw water temperature increased by +0.3 °C annually at both study sites (Figure 3 (3,4)).

**Figure 3:**
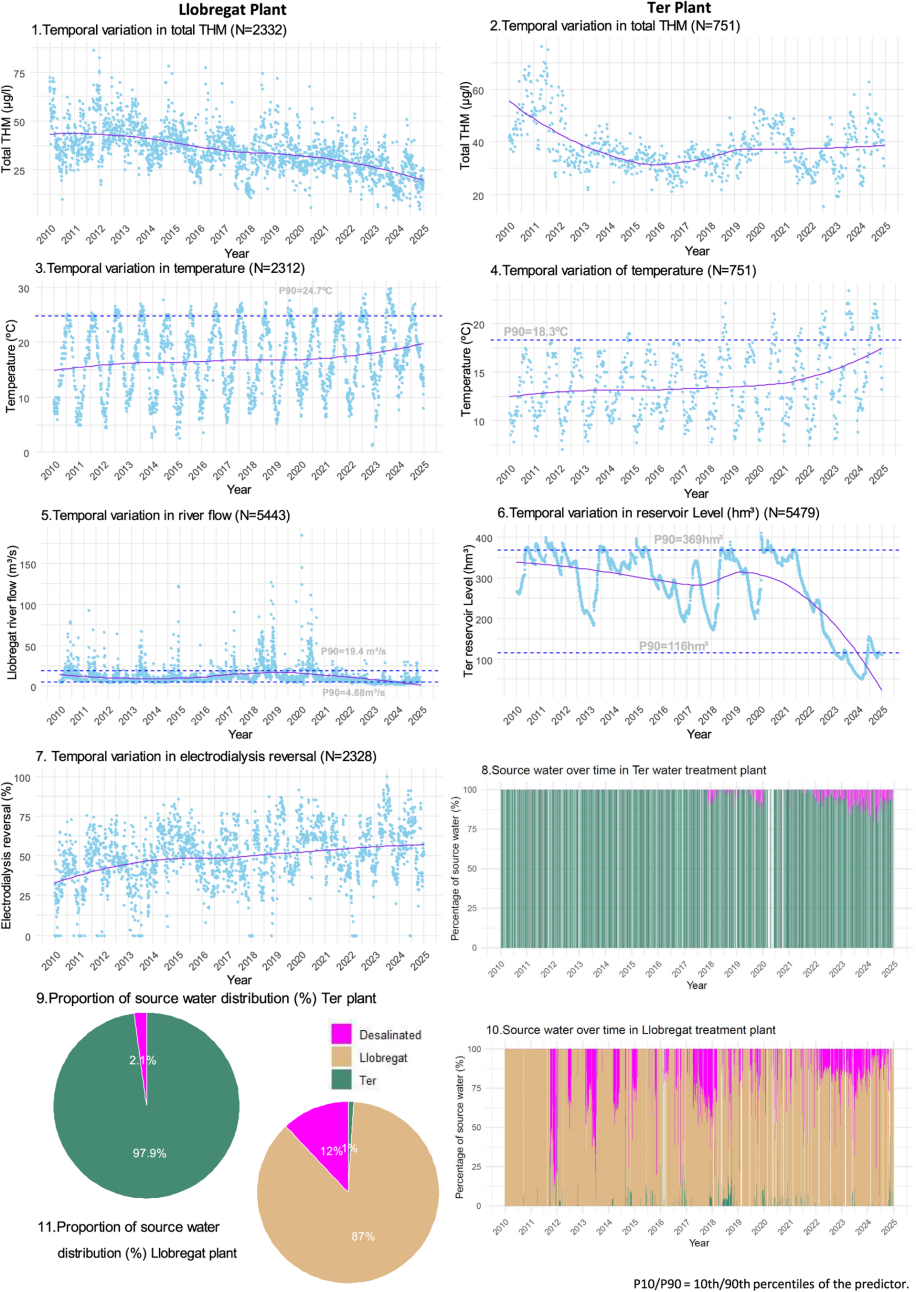
Time-series of total trihalomethanes (THMs), temperature, river flow, reservoir level, and electrodialysis reversal. The proportion of water source in both treatment plants.

**Table 1:** Descriptive Statistics of the Main Variables under This Study

			Llobregat plant		Ter plant	
parameters			N	median (P25, P75)	N	median (P25, P75)
Chloroform (CHCl3, μg/L)			2331	<0.5 (<0.5, 1.4)	751	23.0 (19.0, 24.7)
Bromodichloromethane (CHCl2Br, μg/L)			2331	3.0 (2.0, 4.4)	751	8.8 (7.8, 10.0)
Dibromochloromethane (CHClBr2, μg/L)			2331	10.5 (8.0, 13.0)	751	2.6 (2.0, 3.7)
Bromoform (CHBr3, μg/L)			2331	17.4 (13.0, 22.9)	751	<0.5 (<0.5, < 0.5)
Total trihalomethane (THM4, μg/L)			2331	34.3 (27.8, 41.7)	751	35.8 (31.4, 42.1)
**Predictors**						
continuous	temperature (°C)		2312	17.0 (12.0, 22.0)	751	13.4 (11.0, 16.4)
	river flow (m^3^/s)		5443	8.3 (6.5, 11.8)	-	-
	reservoir volume (Hm^3^)		-	-	5479	305 (217, 348)
	standardized precipitation evapotranspiration index (SPEI 1)		5445	–0.1 (−0.8, 0.7)	5479	–0.1 (−0.8, 0.7)
categorical			all sources Llobregat (N)	only Llobregat (N)	Llobregat+ Desalinated (N)	all sources Ter (N)
	high temperature (≥P90)		232	76	141	74
	normal temperature (<P90)		2080	1060	766	677
	low river flow/reservoir volume (≤P10)		544	16	207	548
	high river low/reservoir volume (≥P90)		545	95	50	548
	normal river flow/reservoir volume (>P10, <P90)		4354	1038	653	4383
	SPEI 1 ≤ −1.5 (severe drought)		271	42	64	376
	SPEI 1 ≥ 1.5 (severe wet)		389	71	73	369
	SPEI 1 < |1.5|		4782	1036	773	4734

			Llobregat plant		ter plant	
**Covariates**			N	Median (P25, P75)	N	Median (P25, P75)
Conductivity (μS/cm)			2317	1331 (1166, 1491)	749	408 (388, 433)
Total organic carbon (TOC, mg/L)			2299	3.2 (2.8, 3.7)	749	2.6 (2.4, 3.0)
Ammonia (mg/L)			2331	0.1 (0.1, 0.2)	757	0.1 (0.1, 0.2)
Nitrate (mg/L)			2309	7.5 (5.9, 9.7)	751	4.2 (<0. 5, 6.0)
pH			2315	8.1 (8.0, 8.2)	749	8.0 (7.9, 8.0)
**Operational Variables**						
electrodialysis reversal (EDR, %)			2219	40.8 (39.4, 62.0)	-	-
desalinated water (%)			1048	22.0 (14.7, 34.3)	219	6.6 (4.2, 9.6)
Ter water (%)			272	6.0 (3.7, 10.5)	-	-

### Llobregat Plant

River flow showed a nonlinear association with THM concentrations, with distinct patterns depending on the THM species. Temperature was positively associated with most THM species [THM4 by 0.24 (CI 95%: 0.08, 0.39)] except for CHCl2Br, which showed no significant associations (Figure 4).

**Figure 4:**
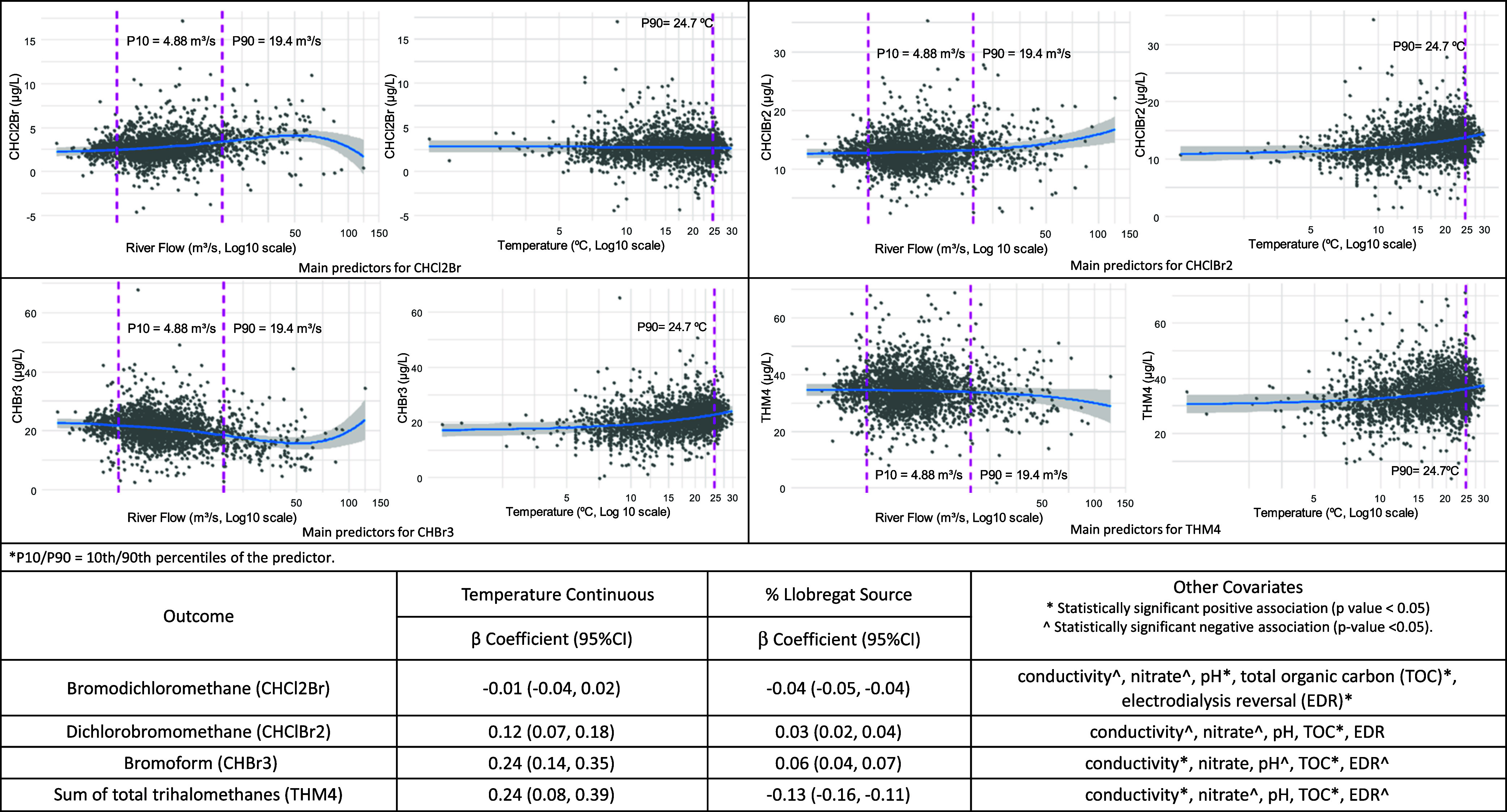
Trihalomethanes change in the Llobregat plant by river flow and temperature in continuous, based on a generalized additive model adjusted for time (spline), water source, and covariates.

Extreme high temperature (≥P90, 24.7 °C) did not significantly affect THM (Table 2) overall, except for a positive association with bromoform for the subset including only Llobregat water (β: 1.41, 95%CI: 0.13, 2.69). However, high river flow (P90 ≥ 19.4 m^3^/s) was inversely associated with THM4 (β: −1.41, CI 95%: −2.77, −0.05) and CHBr3 (β: −2.64, CI 95%: −3.61, −1.67) but positively associated with CHClBr2 (β: 0.79, CI 95%: 0.28, 1.29) and CHCl2Br (β: 0.74, 95%CI: 0.46, 1.02).

**Table 2:** Change (β, 95% confidence interval-CI-) in Trihalomethanes Concentrations by River Flow or Reservoir Volume and Temperature Extreme Percentiles or by Standard Precipitation–Evapotranspiration Index (SPEI) Extremes

			river flow/reservoir volume				SPEI 1	
analysis group by water source	outcome^a^	N	low (≤P10) β (95%CI)	high (≥P90) β (95%CI)	temperature high (≥P90) β (95% CI)	N	≤-1.5 (severe drought) β (95%CI)	≥1.5 (severe Wet) β (95%CI)
Llobregat plant, all data^b^	CHCl2Br	2262	0.32 (−0.02, 0.65)	0.74 (0.46, 1.02)	–0.15 (−0.41, 0.11)	2274	–0.06 (−0.38, 0.25)	0.52 (0.24, 0.79)
	CHClBr2		0.19 (−0.42, 0.80)	0.79 (0.28, 1.29)	–0.34 (−0.80, 0.13)		–0.03 (−0.59, 0.54)	0.59 (0.10, 1.09)
	CHBr3		–0.37 (−1.54, 0.81)	–2.64 (−3.61, −1.67)	0.30 (−0.59, 1.20)		0.09 (−1.00, 1.18)	–1.11 (−2.06,–0.16)
	THM4		0.09 (−1.55, 1.74)	–1.41(−2.77, −0.05)	–0.25 (−1.51, 1.01)		–0.78 (−2.31, 0.74)	0.91(−0.42,2.24)
Llobregat plant, only Llobregat River^c^	CHCl2Br	1119	0.02 (−0.64, 0.68)	1.22 (0.96, 1.49)	–0.11 (−0.40, 0.17)	1125	–0.31 (−0.66, 0.04)	0.59 (0.30, 0.87)
	CHClBr2		–0.40 (−2.09, 1.29)	0.92 (0.25, 1.59)	0.01 (−0.73, 0.75)		–0.30 (−1.18, 0.58)	1.37 (0.66, 2.07)
	CHBr3		–2.60 (−5.53, 0.33)	–3.46 (−4.63, −2.29)	1.41 (0.13, 2.69)		–0.04 (−1.60, 1.51)	–0.51 (−1.76, 0.74)
	THM4		–3.08 (−7.13, 0.98)	–0.77 (−2.38, 0.84)	1.36 (−0.41, 3.12)		–0.60 (−2.71, 1.51)	1.69 (−0.01, 3.39)
Llobregat plant, river + desalinated^c^	CHCl2Br	890	0.23 (−0.17, 0.62)	–0.05 (−0.64, 0.54)	0.18 (−0.21, 0.58)	891	0.25 (−0.28, 0.78)	0.73 (0.27, 1.18)
	CHClBr2		0.20 (−0.43, 0.82)	–0.52 (−1.47, 0.43)	–0.07 (−0.70, 0.57)		–0.03 (−0.92, 0.86)	0.01 (−0.72, 0.74)
	CHBr3		0.58 (−0. 77, 1.93)	–2.81 (−4.85, −0.76)	–0.40 (−1.77, 0.97)		–0.44 (−2.32, 1.43)	–2.42 (−3.98, −0.87)
	THM4		1.32 (−0.48, 3.12)	–4.36 (−7.08, −1.64)	0.13 (−1.70, 1.95)		–0.36 (−2.85, 2.14)	–0.09 (−2.19, 2.01)
Ter plant, all data^d^	CHCl3	745	2.99 (0.29, 5.69)	1.63 (0.33, 2.92)	1.56 (−0.05, 3.18)	745	–1.78 (−3.10, −0.46)	–1.74 (−3.15, −0.33)
	CHCl2Br		1.35 (0.67, 2.04)	0.10 (−0.23, 0.43)	0.33 (−0.08, 0.75)		0.004 (−0.34, 0.35)	–0.20(−0.57, 0.16)
	CHClBr2		0.59 (0.05, 1.13)	–0.04 (−0.30, 0.21)	–0.21 (−0.53, 0.11)		0.15 (−0.11, 0.41)	–0.10 (−0.37, 0.18)
	THM4		4.08 (0.83, 7.33)	1.64 (0.09, 3.19)	0.87 (−1.06, 2.81)		–1.82 (−3.39, −0.24)	–2.15 (−3.83, −0.46)

a CHCl3 = chloroform, CHCl2Br = bromodichloromethane, CHClBr2 = dibromochloromethane, CHBr3 = bromoform, THM4 = sum of all f the trihalomethane.

b Adjusted for source, conductivity, ammonia, pH, total organic carbon (TOC), and % electrodialysis reversal (EDR).

c Adjusted for conductivity, ammonia, pH, TOC, and %EDR.

d Adjusted for source, conductivity, nitrate, pH, and TOC.

Stratification by a specific water source showed evidence of effect modification in some THM species. CHCl2Br and CHClBr2 were positively associated with river flow when water was 100% from the Llobregat river but showed no association among the subset, including Llobregat and desalinated water. Similar case for THM4, the negative association was stronger in the mixed-source group, suggesting an effect modification. In contrast, the negative association between high river flow and CHBr3 remained consistent across both groups, indicating no effect modification (Table 2).

According to SPEI 1, severe wet conditions were positively associated with higher CHCl2Br and CHClBr2 concentrations, β: 0.52 (CI 95%: 0.24, 0.79) and β: 0.59 (CI 95%: 0.10, 1.09), respectively, and negatively associated with CHBr3, β: −1.11 (CI 95%: −2.06, −0.16) (Table 2). The magnitude of the association was stronger than the hydrological parameter (high river flow). Stratification by a water source confirmed consistent patterns for chlorinated THMs. However, no association was found under severe drought conditions.

### Ter Plant

Temperature as a continuous variable was positively associated with most THMs, including THM4, increasing by 0.35 (CI 95%: 0.07, 0.63), except for CHClBr2 (Figure 5). Reservoir levels also showed a linear relationship with the THM species.

**Figure 5:**
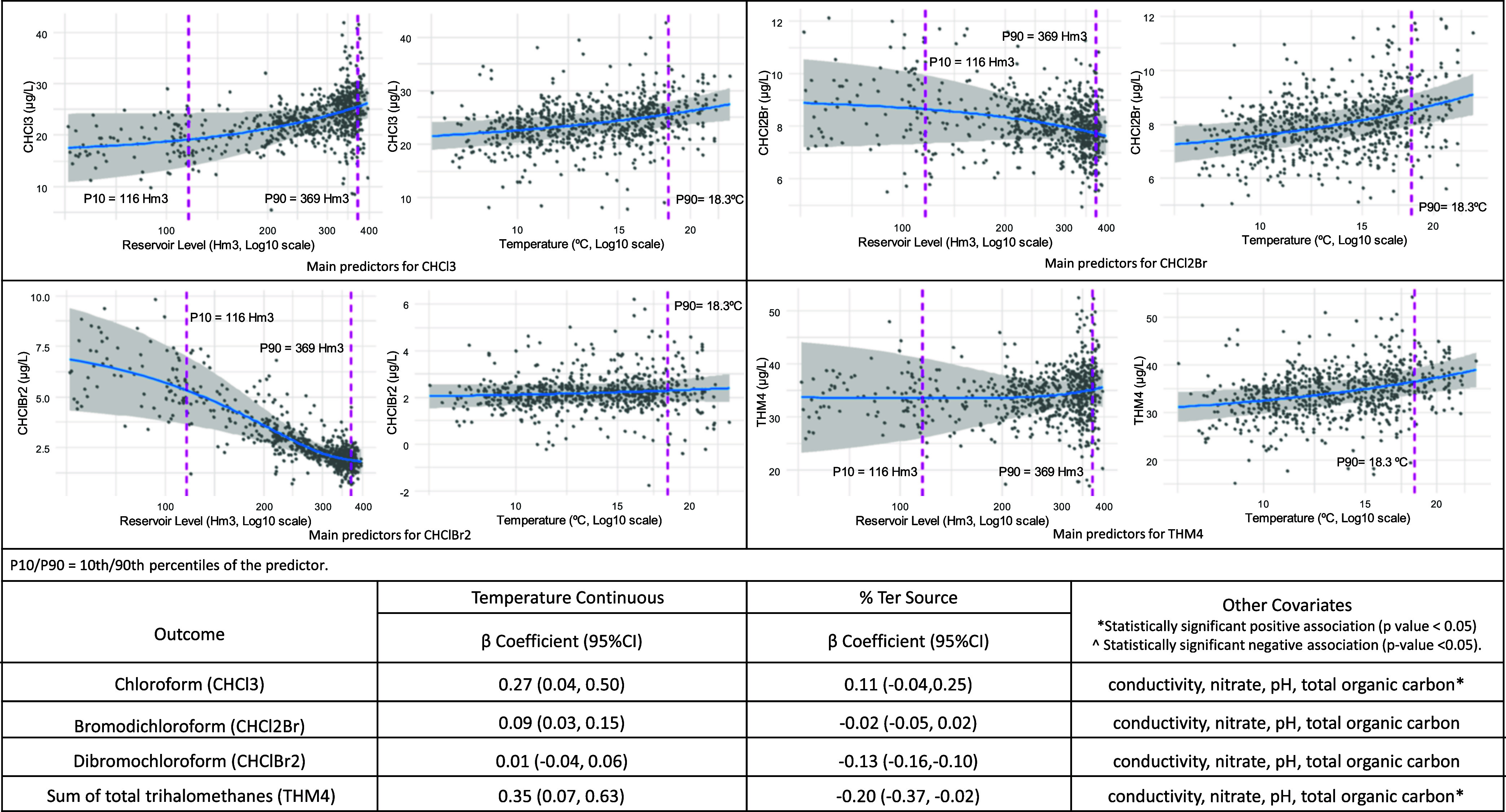
Trihalomethanes change in the Ter plant by reservoir level and temperature in continuous, based on a generalized additive model adjusted for time (spline), water source, and covariates.

Extreme high temperatures (≥P90, 18.3 °C) did not show a significant association with THM species (Table 2). In contrast, low reservoir levels (≤P10, 116 hm3) significantly increased concentrations in all THM species, most notably CHCl3 by 2.99 (CI 95%: 0.29, 5.69) and THM4 by 4.08 (CI 95%: 0.83, 7.33). High reservoir levels (P90 ≥ 369 hm^3^) also increased THM formation, such as CHCl3 by 1.63 (CI 95%: 0.33, 2.92) and THM4 by 1.64 (CI 95%: 0.09, 3.19).

According to the SPEI 1 index, THM4 levels declined significantly during both severe drought and wet conditions by −1.82 (CI 95%: −3.39, −0.24) and −2.15 (CI 95%: −3.83, −0.46), respectively, compared to normal conditions. However, when examining consistency, the hydrological results represented by reservoir volume were not aligned with the SPEI 1 index (Table 2).

## Discussion

This study assessed the influence of extreme hydrologic and weather events on THM formation in two contrasting drinking water production systems in the Barcelona Metropolitan Area: a river-fed system (the Llobregat plant) and a reservoir-based system (the Ter plant). By combining long-term monitoring with event-based modeling, we identified how certain extreme hydrometeorological events, along with operational factors, modulate both THM formation and speciation. Over the 15-year study period, THM4 levels declined across both plants, reflecting operational upgrades and improved treatment efficiency. Notably, the overall influence of extreme events on THM concentration appeared modest in magnitude; however, the shift in speciation is the key finding.

The Llobregat plant, a dynamic riverine system with highly flexible operational capabilities such as extensive source blending and EDR technology, demonstrates substantial adaptability to extreme hydrometeorological conditions. High river flow events were associated with reduced levels of brominated THMs (e.g., CHBr3), while at the same time an increase of chlorinated species such as CHCl2Br and CHClBr2 was observed. These effects persisted after accounting for EDR and the water source. The speciation shift might reflect precursor dilution (lower bromide) and increased TOC from surface runoff, particularly when exclusively Llobregat river water is treated and therefore potentially influences THM levels beyond the buffering capacity of operational strategies. The shift toward less brominated species also implies a lower presence of more toxic THM, which may have positive implications for public health, considering that more brominated species are generally regarded as more hazardous.^26^ A key operational factor would be the use of EDR, contributing not only to the reduction of the overall THM4 but also to the shift in speciation toward less brominated THMs. The ability of EDR to consistently lower bromide levels suggests its strategic value in mitigating climate-sensitive formation pathways, particularly those driven by high temperature and elevated bromide availability.^16^ The composition of the source water acted as a significant effect modifier. When desalinated seawater was blended with river water, the association was notably attenuated between high flow and increased levels of chlorinated THM species. This highlights the role of source water management in buffering treatment plants against fluctuations in natural hydroclimatic events and how important it is to account for blending regimes in risk modeling. In contrast, low river flow events showed no significant effect on the THM levels. This lack of effect may be attributable to the combined buffering capacity of EDR and the strategic use of desalinated water during dry periods, both of which reduce the bromide content and thereby decouple THM formation from temperature-driven kinetics. As a result, the relatively weak temperature effect observed in our models likely reflects successful operational decoupling of key chemical precursors from the environmental conditions.

The Ter plant, supplied by a system of interconnected reservoirs, is operationally less complex than the Llobregat system. However, like the Llobregat plant, the continuous temperature model showed a significant association with THM formation, while the extreme temperature events model did not. This discrepancy suggests that gradual and sustained temperature increases might be more influential than short-term extremes. Noted in previous literature, effects of extreme weather events vary depending on their duration, timing, and the intensity,^41^ which may explain the lack of consistent patterns under binary extreme event classification. Both low and high extreme reservoir volumes were significantly associated with increased THM formation, likely through different pathways. Low reservoir volumes may lead to concentrated organic precursors, enhancing THM formation. Notably, most of those extreme low reservoir events were registered over the recent years, coinciding with periods where blending desalinated seawater system was mostly used and therefore altering water composition. Conversely, extreme high reservoir volume was registered over the 15-year period, associated with increased THM formation. These episodes of high-water inflow, whether from storm-driven events, snowmelt, or upstream releases, may contribute to the mobilization of organic matter from runoff and can also induce vertical mixing within the reservoir.^42,43^ Such mixing can bring organic matter and other compounds from deeper layers or resuspend accumulated material from the reservoir bottom, leading to increased TOC levels and other precursors of THM. Both extremes pose potential risks to source water quality through different pathways. However, it is important to note that within the interconnected three-reservoir system, raw water quality is routinely monitored at multiple depths in the two upstream reservoirs. This monitoring informs the selection of intake gates, allowing operators to choose the water layer with the most favorable quality before it is transferred to the final reservoir, functioning as a large mixing basin prior to treatment in the drinking water plant.

The SPEI 1 index showed associations with THM species, particularly in the Llobregat plant, suggesting it may serve as a proxy for climate influence in river-based systems in short-term extreme weather events. To assess the robustness of this finding, we tested a longer period using SPEI 3 and applied time lags to the SPEI 1 index. In both cases, the models yielded similar or lower adjusted R-squared values or lost statistical power due to the reduction of sample size (Supporting Table 1). In the Ter plant, associations between SPEI 1 and THM were less aligned with hydrological measures, likely due to the buffering effect of reservoir storage. Longer-term indices (such as SPEI 3 or SPEI 6) would better capture long-term meteorological variations, but neither is appropriate for short-term extreme weather events.

We acknowledge several limitations that may have impacted the estimation of extreme weather effects on the THM formation. The use of a high-order spline allowed us to model nonlinear responses and preserve data granularity. However, it may have attenuated some of the variability attributable to extreme weather events, limiting our ability to detect their independent effect; this could help explain the relatively small effect attributed to hydrological parameters. The modest effect may reflect either a true buffering effect of treatment systems or methodological constraints in detecting episodic impacts within a smoothed temporal framework. Additionally, the temporal resolution of the data, with sampling occurring every 4–5 days to weekly, may have constrained our ability to fully capture rapid changes during short-duration extreme events. Mediterranean weather is characterized by flash floods, and it is already reported in the literature that even if there is an initial phase of increasing concentrations of TOC and other matters by runoff, the water quality during the events could also change. Therefore, this limitation reduces the sensitivity of the models. Although collinearity was not detected, high correlation between key variables warrants cautious interpretation such as between Ter River water percentage and reservoir volume (*r* = −0.63) (Supporting Figure 1). The interaction may have enhanced the individual effect of those variables on the final model of THM formation. However, the inclusion of those variables was essential for predicting THM, and including the interaction effect in the models made no difference. Finally, other operational factors such as chlorine dose, contact time, and residence time were not considered, given that we focused on THM analysis at the outlet of the treatment plants, prior to distribution. Here, the chlorine dose is controlled to keep the chlorine residue under stable concentrations; thus, chlorine dose varies minimally over time, and residence or contact time within the distribution network is not relevant case. Although residence time during storage could be relevant, this tends to be highly stable.

Future research should also consider postdrought rewetting events, which may have triggered increased TOC levels and subsequent THM formation,^44^ highlighting the need to view extreme weather impacts not as an isolated episode but within a broader hydrological context. To better capture these dynamics, we recommend the use of complementary modeling approaches, such as seasonal-trend decomposition, random forest models, generalized additive mixed models, or weighted regression techniques, paired with high-frequency monitoring.^29^ These could enhance the detection of complex interactions between extreme weather events, source water quality, and treatment operations.

## Conclusion

This study applied a long-term analysis in two contrasting drinking water plants to assess how extreme weather events influence THM formation and speciation. Overall, the effects of extreme conditions on THM levels were modest; however, distinct patterns emerged, depending on water source and treatment configuration. The Llobregat plant, with its flexible use of EDR treatment and source mixing, appeared to buffer THM formation under extreme weather events. In contrast, the Ter system, reliant on reservoir dynamics such as selective withdrawal and artificial sedimentation cell combining three reservoir systems with less operational flexibility, showed greater sensitivity to volume extremes. These findings highlight the importance of integrating source water characteristics, advanced treatment technologies, and long-term monitoring to improve climate resilience in drinking water systems. Future research should aim to capture short-term events with higher-frequency data and explore broader operational variables to enhance the predictive capacity. Studies in other settings with less intervened drinking water production systems are needed to understand the effect of extreme weather events on drinking water quality.

## Supplementary Material



## Notes

Spearman correlation matrix among the hydrometeorological variables used in the study (Figure 1); the results of the model using alternative SPEI periods (SPEI 3) and lagged SPEI 1 index to evaluate the robustness of the main model (Table 1) (PDF)
