# Supplementary material for: Impact of Climate and Hydrological Variability on Drinking Water Production and Trihalomethane Levels: A Case Study in Barcelona, Spain (2010–2024)
Source: ACS ES T Water. 2025 Nov 25;5(12):7524–35. doi: 10.1021/acsestwater.5c01024 (PMC12706784; doi:10.1021/acsestwater.5c01024)
Supplement: Supplementary file 1 [file ew5c01024_si_001.pdf]

Supporting information for:

Impact of climate and hydrological variability on drinking water production and trihalomethane levels. A case-study in Barcelona, Spain (2010-2024).

Fang Fang Chen Chen[a,b], Pere Emiliano[c], Fernando Valero[c], Xavier Basagaña[a,b,d,e], Cristina M Villanueva[a,b,d,e]*

a) ISGlobal, Doctor Aiguader 88, Barcelona 08003, Spain.
b) Universitat Pompeu Fabra (UPF), Doctor Aiguader 88, Barcelona 08003, Spain.
c) Ens d'Abastament d'Aigua Ter Llobregat (ATL) Sant Martí de l´Erm, 2. 08970 Sant Joan Despí, Barcelona, Spain.
d) CIBER Epidemiologia y Salud Pública (CIBERESP), Instituto de Salud Carlos III, Madrid 28029, Spain.
e) Hospital del Mar Medical Research Institute, Doctor Aiguader 88, Barcelona 08003, Spain.

* Email: cristina.villanueva@isglobal.org

Supplementary Table 1. Change in trihalomethanes concentrations by standard precipitation evapotranspiration index (SPEI) extremes by month and by trimester

**Analysis group by water source**	**Outcome[1]**	**N**	**R^2 Adj**	**SPEI 3**		**R^2 Adj**	**SPEI 1**		**N**	**R^2 Adj**	**SPEI 1 lag 7**	
				**≤-1.5 (Severe Drought) β (95%CI)**	**≥ 1.5 (Severe Wet) β (95%CI)**		**≤-1.5 (Severe Drought) β (95%CI)**	**≥ 1.5 (Severe Wet) β (95%CI)**			**≤-1.5 (Severe Drought) β (95%CI)**	**≥ 1.5 (Severe Wet) β (95%CI)**
**Llobregat plant, all data[2]**	**CHCl2Br**	2274	0.56	- 0.21 (- 0.52, 0.10)	- 0.01 (-0.41,0.39)	0.56	-0.06 (-0.38, 0.25)	0.52 (0.24, 0.79)	1989	0.47	-0.002 (-0.37, 0.37)	1.08 (0.75, 1.41)
	**CHClBr2**		0.59	-0.76 (-1.32, -0.21)	0.75 (0.03, 1.46)	0.59	-0.03 (-0.59, 0.54)	0.59 (0.10, 1.09)		0.55	-0.92 (-1.55, -0.28)	1.47 (0.90, 2.05)
	**CHBr3**		0.52	-0.71 (-1.77, 0.36)	-0.37 (-1.74, 0.99)	0.52	0.09 (-1.00, 1.18)	-1.11 (-2.06,-0.16)		0.50	-1.08 (-2.29, 0.12)	-1.42 (-2.50, -0.33)
	**THM4**		0.61	-2.21 (-3.71, -0.71)	-0.47 (-2.40,1.45)	0.56	-0.78 (-2.31, 0.74)	0.91(-0.42,2.24)		0.56	-1.11 (-2.84, 0.61)	2.48 (0.93, 4.04)
**Llobregat plant, only Llobregat River[3]**	**CHCl2Br**	1125	0.53	- 0.16 (-0.54, 0.21)	0.51 (0.13, 0.89)	0.54	-0.31 (-0.66, 0.04)	0.59 (0.30, 0.87)	1118	0.39	-0.12 (-0.52, 0.28)	0.19 (-0.14, 0.52)
	**CHClBr2**		0.54	-0.41 (-1.34, 0.52)	1.55 (0.61, 2.49)	0.54	-0.30 (-1.18, 0.58)	1.37 (0.66, 2.07)		0.44	-0.34 (-1.30, 0.61)	1.20 (0.42, 1.98)
	**CHBr3**		0.64	-0.72 (-0.94, 2.39)	-0.92 (-2.60, 0.77)	0.64	-0.04 (-1.60, 1.51)	-0.51 (-1.76, 0.74)		0.60	1.64 (-0.002, 3.27)	3.32 (1.98, 4.67)
	**THM4**		0.61	-0.11 (-2.37, 2.15)	1.08 (-1.20, 3.36)	0.61	-0.60 (-2.71, 1.51)	1.69 (-0.01, 3.39)		0.58	1.02 (-1.17, 3.22)	4.60 (2.80, 6.39)
**Llobregat plant, river + desalinated[3]**	**CHCl2Br**	891	0.73	-0.45 (-0.95, 0.06)	-0.51 (-1.35, 0.32)	0.73	0.25 (-0.28, 0.78)	0.73 (0.27, 1.18)	885	0.67	-0.07 (-0.69, 0.55)	0.82 (0.31, 1.34)
	**CHClBr2**		0.65	-0.67 (-1.51, 0.18)	-1.38 (-2.70, -0.05)	0.65	-0.03 (-0.92, 0.86)	0.01 (-0.72, 0.74)		0.51	-0.14 (-1.21, 0.93)	1.63 (0.76, 2.49)
	**CHBr3**		0.37	-0.61 (-2.38, 1.16)	-2.19 (-5.04, 0.67)	0.38	-0.44 (-2.32, 1.43)	-2.42 (-3.98, -0.87)		0.33	0.26 (-1.83, 2.35)	-0.65 (-2.30, 1.00)
	**THM4**		0.74	-3.16 (-5.53, -0.78)	-5.14 (-8.96, -1.33)	0.73	-0.36 (-2.85, 2.14)	-0.09 (-2.19, 2.01)		0.69	0.66 (-2.21, 3.53)	1.32 (-0.98, 3.62)
**Ter plant, all data[4]**	**CHCl3**	745	0.79	-0.99 (-2.43, 0.45)	-0.60 (-2.25, 1.04)	0.79	-1.78 (-3.10, -0.46)	-1.74 (-3.15, -0.33)	705	0.78	-0.44 (-1.86, 0.98)	-1.96 (-3.48, -0.43)
	**CHCl2Br**		0.64	0.09 (-0.28, 0.47)	0.01 (-0.42, 0.43)	0.64	0.004 (-0.34, 0.35)	-0.20(-0.57, 0.16)		0.63	0.15 (-0.20, 0.50)	-0.24 (-0.62, 0.14)
	**CHClBr2**		0.87	0.08 (-0.21, 0.36)	0.05 (-0.27, 0.37)	0.87	0.15 (-0.11, 0.41)	-0.10 (-0.37, 0.18)		0.86	-0.005 (-0.28, 0.27)	-0.14 (-0.44, 0.16)
	**THM4**		0.73	-0.51 (-2.24, 1.22)	-0.54 (-2.51, 1.43)	0.74	-1.82 (-3.39, -0.24)	-2.15 (-3.83, -0.46)		0.73	-0.59 (-2.27, 1.10)	-2.41 (-4.22, -0.61)

[1]CHCl3 = chloroform, CHCl2Br = bromodichloromethane, CHClBr2 = dibromochloromethane, CHBr3 = bromoform, THM4 = Sum of all the trihalomethane

[2]Adjusted for source, conductivity, ammonia, pH, TOC, and %EDR.

[3]Adjusted for conductivity, ammonia, pH, TOC, and %EDR.

[4]Adjusted for source, conductivity, nitrate, pH, and TOC.

a. Hydrological variables

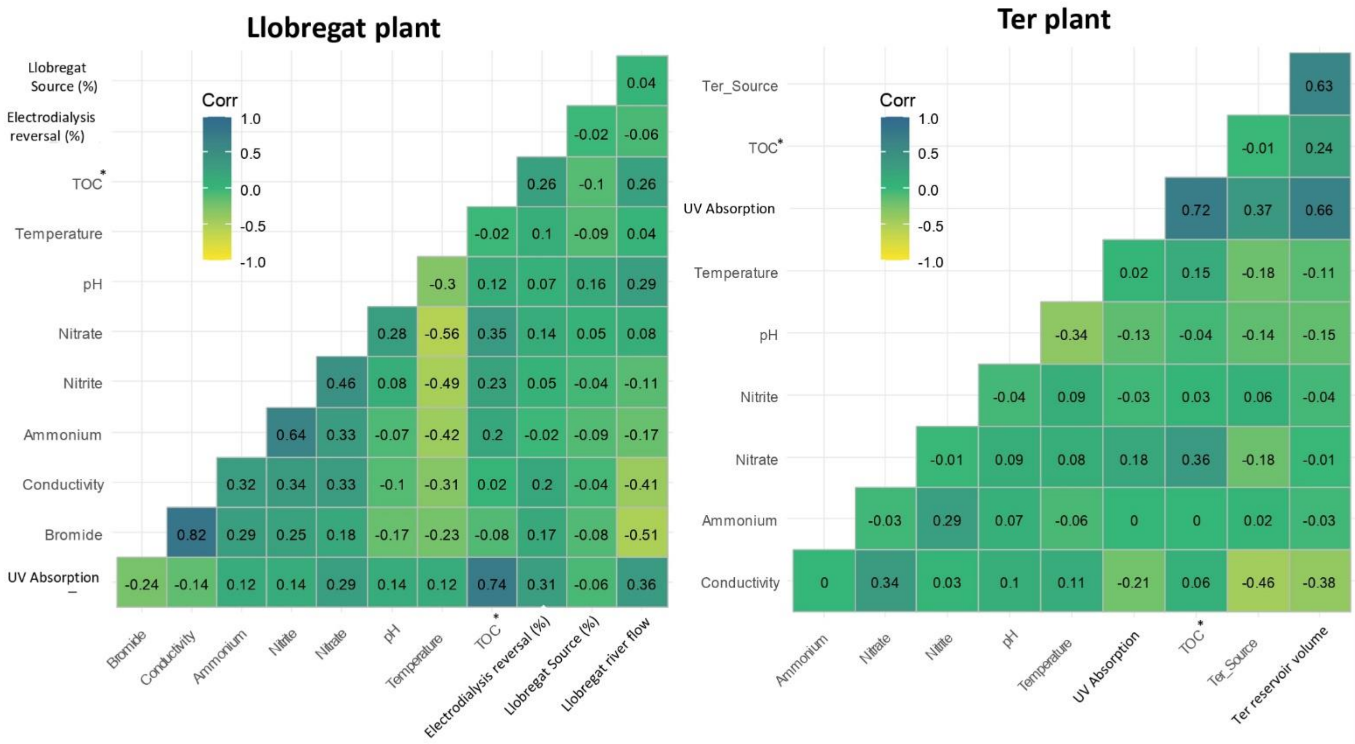

*TOC, Total Organic Carbon

b. Hydrological covariates and meteorological SPEI- 1 Index

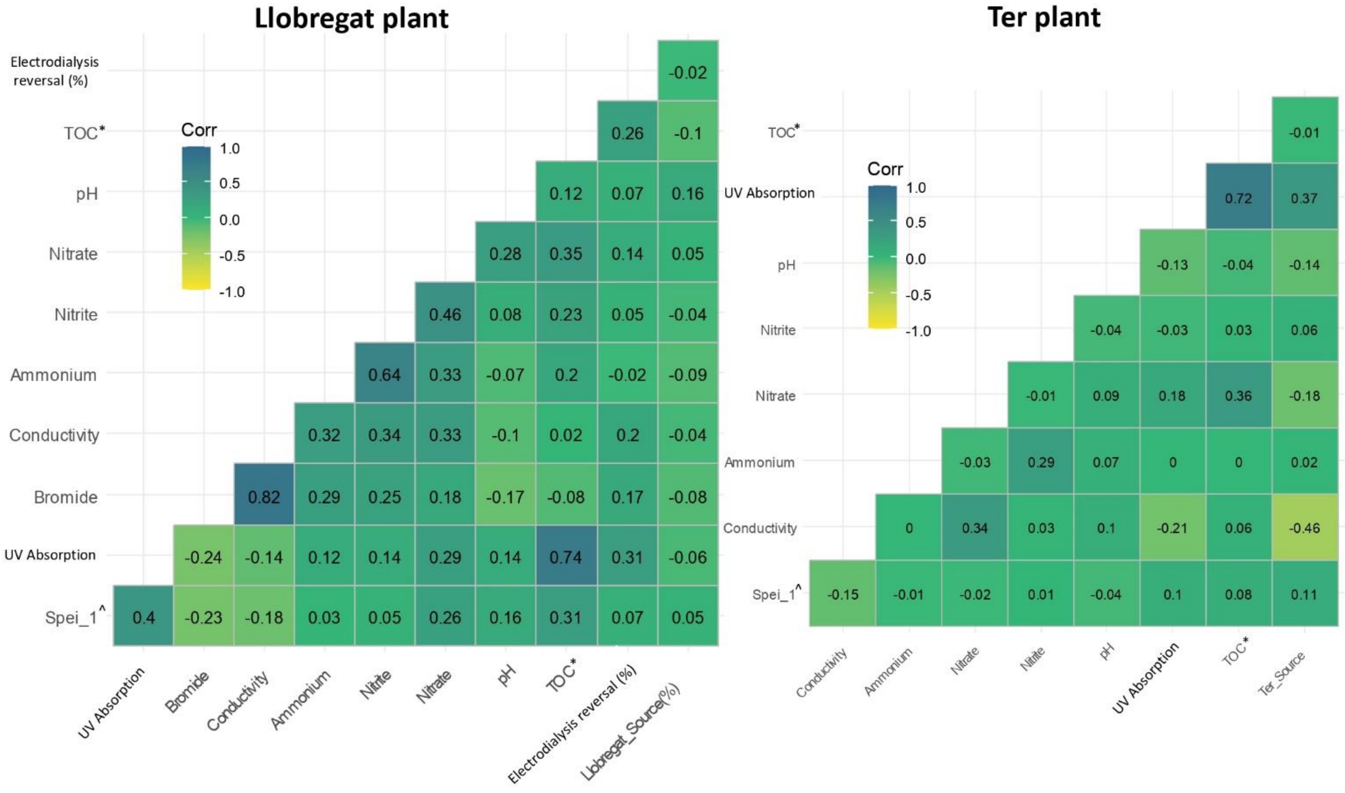

*TOC, Total Organic Carbon
^Spei_1, Standardized Precipitation Evapotranspiration Index

SUPPLEMENTARY FIGURE 1. Spearman correlation plots of hydrometereological variables
